# Indoor Temperature Prediction in an IoT Scenario

**DOI:** 10.3390/s18113610

**Published:** 2018-10-24

**Authors:** Pedro Lima Monteiro, Massimiliano Zanin, Ernestina Menasalvas Ruiz, João Pimentão, Pedro Alexandre da Costa Sousa

**Affiliations:** 1 Department of Electrical and Computer Engineering, Faculty of Science and Technology, Universidade Nova de Lisboa, 2829-516 Lisboa, Portugal; pim@fct.unl.pt (J.P.); pas@fct.unl.pt (P.A.d.C.S.); 2 Centro de Tecnologías Biomédicas, Universidad Politécnica de Madrid, 28223 Madrid, Spain; massimiliano.zanin@ctb.upm.es (M.Z.); ernestina.menasalvas@upm.es (E.M.R.)

**Keywords:** Internet of Things, temperature sensors, home automation

## Abstract

One of the hottest topics being researched in the field of IoT relates to making connected devices smarter, by locally computing relevant information and integrating data coming from other sensors through a local network. Such works are still in their early stages either by lack of access to data or, on the other hand, by the lack of simple test cases with a clear added value. This contribution aims at shading some light on how knowledge can be obtained, using a simple use case. It focuses on the feasibility of having a home refrigerator performing temperature forecasts, using information provided by both internal and external sensors. The problem is reviewed for both its potential applications and to compare the use of different algorithms, from simple linear correlations to ARIMA models. We analyse the precision and computational cost using real data from a refrigerator. Results indicate that small average errors, down to ≈0.09 °C, can be obtained. Lastly, it is devised how can the scenario be improved, and, most importantly, how this work can be extended in the future.

## 1. Introduction

The introduction of the Internet of Things (IoT) paradigm is dramatically transforming the philosophy behind the design and operation of many devices [1,2,3]. These devices were once isolated elements, which could only rely on themselves and on their internal resources; now, they can form a network sharing both resources and information. As an example, just a few years ago, if one device needed some specific information about the environment, the only possible solution entailed including the corresponding sensor (and all its controlling logic) within the device itself. This implied a two-fold consequence: the additional cost associated with the new sensors, and the fact that non-essential information was seldom gathered. The IoT radically changed this state of affairs: the same device can now wirelessly communicate with other devices, and receive from them any (or part of the) information they customarily collect.

To better illustrate the significance of the changes introduced by this paradigm, let us focus on the specific example of measuring and forecasting the temperature inside a building. The knowledge of the present and future indoor temperature has many potential applications. First of all, on a meso scale, it can be used to forecast the building energy consumption, which in turn can result in a more efficient energy management (and thus in a reduction of its environmental impact) [4,5]. Information coming from different buildings can then be aggregated at a macro scale, to forecast the energy consumption of a city or of a country [6]. Down to a more micro scale, individual appliances could finally use this information to optimise their internal dynamics. For instance, refrigerators can anticipate changes in the temperature in order to minimise internal variations; or participate in energy markets and arbitrage, and combine short-term environmental forecasts with price projections to reduce operating costs [7]. Additionally, refrigerators can also use this information to forecast their future energy needs, and drive the dynamics of a connected smart grid [8,9].

In the pre-IoT era, this would have required the installation of one temperature sensor per device; additionally, and in order to have a good spatial resolution, the centralised system controlling the building heating would possibly have required one sensor per room. This would have been both expensive and inefficient, as one room may end up containing multiple redundant sensors. IoT technology allows to simplify this scenario, by enabling devices to communicate between them. The temperature recorded by the refrigerator for its own use, and eventually a simple forecast, can be shared with other appliances; and a centralised heating system may use this information as back-up for its own sensor, or as a way of improving the spatial resolution of the information [10,11]. Moreover, indoor temperature can be compared to that of the outdoor sensors, so that even further conclusions can be made and more efficiency can be obtained in the control of all the devices included in an automated household.

While being packed with many advantages, this distributed scenario also presents several challenges. While recording the actual room temperature is a simple task, making a forecast entails a higher cost, both in terms of required memory and computational power. Any solution should thus balance the scenario requirements and the limited resources available inside this kind of devices. Secondly, the information is made available without a central control, implying that a same data element can be provided by multiple devices. Any consumer of such data should then be able to integrate different sources; and, following the previous example, be able to choose among the many sources providing a forecast. Lastly, the distributed nature of IoT requires a synchronisation between the different systems that belong to a given context. This issue is particularly important, as data need to still be relevant at the moment it is being communicated to other devices.

The problem of using machine learning for predicting the indoor temperatures is not new to the literature, with scenarios ranging from individual houses [12,13] to offices and buildings [5,14]. In such literature, the methods vary from the use of Neural Networks to customarily methods and formulas to predict the evolution of indoor temperature according to other features (as heat waves and humidity). Despite this, less attention has been devoted to an IoT context. Some exceptions are [15,16,17], with the majority of the research works found in an IoT context tending to focus on how energy efficiency can be achieved, being indoor temperature used as only a mean instead of an end. Not only that, it was also almost impossible to find, in the literature, any work which used outdoor temperature to predict the indoor one. The sole exception was [18] where the control was simulated using Fuzzy Logic and there is not much indications as to what was used for the actual learning. In this sense, it is possible to conclude that there is a gap in the research where indoor temperature is not predicted using outdoor temperature, especially considering an IoT context. Such prediction can be particularly useful when modelling the functioning of IoT devices whose behaviour depend on the variability of its environment temperature, as it is the case of refrigerators. This prediction ability enables IoT refrigerators to optimise their behaviour using the information regarding its surrounding environment. This very particular gap is, therefore, where this work is inserted in.

In this contribution, we propose a first feasibility study of a scenario in which one appliance measures the environmental temperature, calculates a short-term forecast, and broadcast such information to other devices. For this, we discuss two main challenges. Firstly, while many models have been proposed for predicting the meteorological temperature, much less attention has been devoted to the indoor temperature, as the one that can be recorded in a building. Accordingly, Section 2.1 presents and explains the datasets used for this work, while Section 2.2 presents several alternatives for temperature forecasting, drawn from statistics and statistical physics, which are compared and evaluated in Section 3. Secondly, we discuss the problem of the computational cost, and provide a comparison of each method in Section 3.4. To validate these results, we use a data set representing the real temperature in Lisbon, Portugal, both environmental and indoor. We finally draw some conclusions in Section 4.

## 2. Materials and Methods

### 2.1. Description of the Data Set

The validation of the models that will be described in Section 3 relies on two real data sets. The first one includes the evolution of the indoor temperature recorded by the internal sensor of a refrigerator located in a room at UNINOVA, Lisbon, Portugal. Data correspond to the period from March 2017 to July 2017, with nine values recorded per day—each one of them representing the average temperature for time intervals of approx. 9600 s. The second data set complements this information, by reporting the corresponding outside temperature in the city of Lisbon (more specifically, at the airport of Lisboa Portela), as obtained from the www.wunderground.com/history/ website.

**Figure 1:**
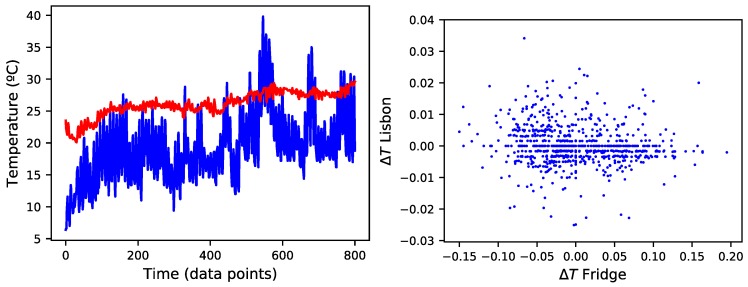
(**Left**) Evolution of the two considered time series, representing the indoor (red line) and outdoor (blue) temperature in Lisbon, Portugal. (**Right**) Evolution of the changes in the outdoor temperature, as a function of the indoor one—see main text for definition.

Both time series are depicted in Figure 1 Left. The 801 observed values span between 20.1 °C to 29.6 °C indoor (average of 26.11, standard deviation of 1.94), and between 6.4 °C and 39.8 °C (average of 19.23, standard deviation of 5.34) outdoor. Additionally, Figure 1 Right depicts a scatter plot representing how changes in the indoor temperature follow those observed outside. Denoting the temperature recorded at time *t* as $T_{t}$, such changes are calculated as:

(1) $$\Delta T_{t}=\log_{10}T_{t+1}/T_{t}.$$

The absence of a clear trend in Figure 1 Right suggests that the evolution of the indoor temperature is largely independent from that of the outside one, due to the building heating/air conditioning system. From the point of view of this work, this suggests that standard temperature forecast models, as the one used in outdoor weather forecasting, may not work when considering indoor temperature.

### 2.2. Algorithms for Temperature Forecasting

Understanding the patterns behind the evolution of temperature, and, hence, its prediction over more or less long periods of time, is a problem whose first scientific solution attempts date back to the beginning of the 20th century [19]. If initial solutions were based on the construction of analytical models (see for instance [20]), the advent of machine learning enabled the development of prediction systems based on historical data (as opposed to expert knowledge). Considered algorithms include neural networks [21,22,23], Support Vector Machines [24] or consensus forecasters [25].

As previously discussed, these models may not be suitable when considering the evolution of the indoor temperature, firstly because the problem is inherently different (i.e., the system to be modelled is the heating/cooling one, and not Nature); and secondly, because of the limitations associated with IoT devices, especially in terms of computational power. To solve this problem, we here consider and describe a set of simple models, which will be the basis to perform a short term forecast.

In the remainder of this section, we review the techniques that have been considered, and that include: linear regression models, data filtering through temperature profile similarities, and ARIMA models. We finally review two naive forecast methods, respectively the mean of the day and the last recorded value, which will be used to validate the models’ performance.

For the sake of clarity, the following notation will be used:

- $T_{i}$: temperature recorded at time *i*.
- *t*: time whose temperature is to be forecast. $T_{t}$ thus represents the temperature to be forecast, and t−1 the moment in time in which the forecast is executed.
- $T_{\left(a:b\right)}$: vector of temperature values between time *a* and *b* (inclusive).

#### 2.2.1. Linear Regression

As a first and basic approach, we have considered the possibility that past and future temperature data may be related by a simple linear relation. As nine values were recorded per day, and one may expect to find some daily periodicities (as, for example, because the heating may always be disconnected at night), a model has been constructed taking into account vectors of 9 historical values. Mathematically, this maps to the following linear model:

(2) $$T_{t}=\beta_{0}+\beta_{1}T_{t-1}+\ldots +\beta_{9}T_{t-9}+\epsilon ,$$

$T_{t}$ being the temperature recorded at time *t* and ϵ a noise term. Using a vector notation, this translates to $T_{t}=\beta T_{\left(t-1:t-9\right)}+\epsilon$. Please note that due to the fact that 9 temperature values are available per day, 8 of them are used to forecast—the remainder one being the target value. Therefore the elements of the parameter vector β encode how the temperature at one moment in time depends on the last day’s values at that same time. The values of β have been obtained through a least squares fit, in each case using the information corresponding to the 30 previous days as training. For the success of such models, it is key that the noise is not correlated with the regressors.

#### 2.2.2. Data Filtering through Networks

**Figure 2:**
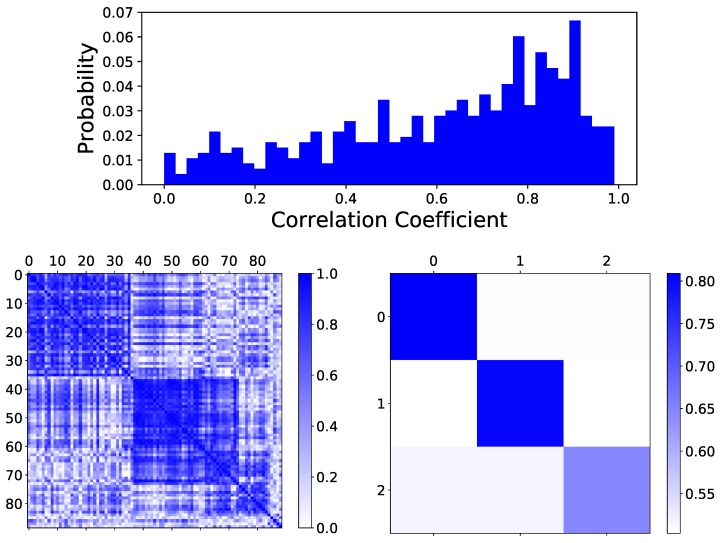
Identification of correlation patterns in temperature data. (**Top**) Histogram of the absolute correlation between all pairs of daily temperature profiles. (**Bottom left**) Correlation matrix between all pairs of daily temperature profiles (i.e., the nodes of the network). Please note that nodes (days) are sorted in order to highlight the community structure. (**Bottom right**) Average correlation within and between communities.

The previously described linear model can be improved if one considers the structure of similarity between days. Specifically, it is plausible to expect some days to share similar characteristics (in this case, temperature profiles), for instance because similar activities are performed in the building; on the contrary, other day pairs may be completely different. To check this hypothesis, Figure 2 Top reports an histogram of the absolute value of the correlation coefficient between pairs of daily temperature profiles. The flatness of the distribution confirms that very different situations can be observed, from pairs containing almost identical to completely uncorrelated days. More importantly, and of relevance for the present work, this result suggests that several days may be eliminated from the training set, as they are not correlated with the target one and are only introducing noise into the model. In other words, the 30 days used to train the linear model of Equation (2) could firstly be analysed and organised in groups; afterwards, only those days belonging to the same group as the target one (and thus presenting a high similarity with it) are used to train the model. The reduction in the quantity of noise introduced in the training should then result in a more effective model—an approach known as *feature selection* in machine learning [26].

To define such groups of days, a complex network approach has been implemented [27]. A functional network has been reconstructed for each value to be forecast, with nodes representing sets of 8 consecutive historical values (thus, the days to be analysed), and links between pairs of them the degree of correlation between the corresponding values. Please note that only 8 values are considered because the target temperature value $T_{t}$ is not known; thus, the vectors associated with nodes, which should also be composed of 8 values, correspond to $\left(T_{t-1},\ldots ,T_{t-8}\right)$, $\left(T_{t-10},\ldots ,T_{t-17}\right)$, and so forth. Afterwards, the absolute value of the Pearson’s linear correlation coefficient between each pair of vectors has been calculated, and stored in a matrix A of size 29×29 (being 29 the number of vectors considered, and the element $a_{i,j}$ the correlation between vectors *i* and *j*). Please note that A is called the *adjacency matrix* of the corresponding network G. Figure 2 Bottom Left depicts an example of such adjacency matrix, where dark blue (respectively, light blue) shades indicate strong (weak) correlations. Thanks to the way nodes are sorted, it is possible to appreciate the existence of communities, or strongly connected groups, which correspond to the blue squares located along the main diagonal.

Subsequently, nodes (thus, vectors of historical temperatures) have been divided in homogeneous groups by applying a *community detection* algorithm [28]. This family of algorithms tries to identify two or more sub-clusters maximising the *modularity* [29], i.e., reaching a situation in which strong connections appear between the nodes within a same module and weak connections only between nodes of different modules. We have specifically used the Python implementation of the Louvain algorithm [30], usually considered one of the most reliable and easily scalable. Figure 2 Bottom represents the resulting adjacency matrix, in which the original 29 nodes have been grouped into three. In this case, the element $a_{i,j}$ of the adjacency matrix represents the average absolute correlation between the elements composing the community *i* and those composing the community *j*. It has to be noted that the reconstructed community structure is meaningful only when the average correlation within one community is always higher than that with other communities—i.e., $a_{i,i}>a_{i,j},\forall j\neq i$. While this is indeed the case in Figure 2 Bottom Right, we checked this condition for all networks through a pair-wise *t*-test (also known as Student’s *t*-test), testing the hypothesis that the correlations between the nodes belonging to a same community and those belonging to different ones come from two distributions of equal average. The resulting *p*-values oscillate between $5.65\times 10^{-113}$ and 0.992, with a 75.37% of them being below the threshold of 0.01, thus confirming the relevance of the obtained communities.

As a final step, a linear model has been trained, as described in Section 2.2.1, only using information of those days that were classified as belonging to the same community as the day to be forecast.

#### 2.2.3. Autoregressive Integrated Moving Average (ARIMA)

Many forecasting methods, such as regressions, rely on the assumption that the time series being forecast are stationary—a situation seldom encountered in real-world problems. When this condition is not fulfilled, but the time series can still be made stationary through differencing, the most general solution entails the use of an autoregressive integrated moving average (ARIMA) model. Such class of models, widely used in econometrics, are usually denoted by the notation ARIMA (p,d,q), where *p* is the order of the autoregressive part, *d* is the order of the differencing part, and *q* is the order of the moving-average part.

Given a time series $\left\{x_{1},x_{2},\ldots \right\}$, the ARIMA model is defined as:

(3) $$\left(1-\sum_{i=1}^{p}\phi_{i}B^{i}\right){\left(1-B\right)}^{d}X_{t}=\mu +\left(1+\sum_{i=1}^{q}\theta_{i}B^{i}\right)\epsilon_{t},$$

*B* being the *back-shift* (or *lag*) operator, defined as $B^{i}X_{t}=X_{t-i}$. Additionally, $\phi_{i}$ and $\theta_{i}$ respectively represent the parameters of the autoregressive and moving average part of the model; μ the average, or drift, of the time series; and $\epsilon_{t}$ error terms. In the sake of synthesis, the ARIMA model can also be expressed through operators as:

(4) $${\left(1-B\right)}^{d}x_{t}=\mu +\frac{\Theta (B)}{\Phi (B)}\epsilon_{t},$$

Φ(B) being the autoregressive operator, defined as $\Phi_{p}\left(B\right)=1-\Phi_{1}\left(B\right)-\ldots -\Phi_{p}{\left(B\right)}^{p}$; and $\Theta_{q}\left(B\right)=1-\Theta_{1}\left(B\right)-\ldots -\Theta_{q}{\left(B\right)}^{q}$ the moving average operator.

Before training the model with historical information, it is necessary to define the value of the parameters *p*, *d* and *q*. Usually this is done by observing the time series, and by calculating the maximum of functions like the Partial AutoCorrelation Function (PACF). This ensures that the chosen parameters are optimal (or close to optimal) for the time series under analysis. Nevertheless, it has to be noted that the limitations in computational power of the IoT devices, where such computations ought to be executed, prevent such optimal solution. As an alternative, we here consider a sub-optimal approximation in which a single set of parameters is calculated *a priori*, using reference historical data. Specifically, all possible parameter values have been tested, between zero and nine, and the set yielding the lowest error retained. Please note that the top has been set to nine for being the number of values describing the temperature vector of one day, and thus being the lag corresponding to the maximum expected autocorrelation.

#### 2.2.4. Kalman Filter

A different approach one can take when it comes to time series forecasting is that of filters. Well known in the engineering world, filters are commonly used to remove spurious components such as noise or vibration, depending on the case study. One of the most used filters in the field of time series analysis is the Kalman filter [31]. Such filter has proven to be useful in almost every situation where there is uncertain information about a system’s dynamics, and an educated guess (the filter’s response) is required about the system’s next state. Its advantages include the ability to extract accurate estimations from noisy time series and its simple implementation. On the other hand, the usage of this type of filter is gaining momentum in IoT contexts, as it requires virtually no memory to perform—all the past dynamics is synthesized within the filter state [32].

The Kalman Filter depends on two tuning parameters: the measurement noise covariance, *R* and the process noise covariance *Q*, which basically relate to how much does the measurement and process change, respectively. On the other hand, and considering a time series $\left\{x_{1},x_{2},\ldots \right\}$, the implementation of this filter depends on five different sets of variables, namely:

- $\hat{x}$—the *a posteriori* estimate of *x*;
- *P*—the *a posteriori* error estimate;
- ${\hat{x}}^{-}$—the *a priori* estimate of *x*;
- $P^{-}$—the *a priori* error estimate;
- *K*—the gain or blending factor, which tends to minimize *P*;

Denoting by $x_{i}$ the time series under analysis, the aforementioned sets relate to each other in the following manners:

(5) $$\begin{array}{c} {\hat{x}}_{i}^{-}={\hat{x}}_{i-1} \end{array}$$

(6) $$\begin{array}{c} P_{i}^{-}=P_{i-1}+Q \end{array}$$

(7) $$\begin{array}{c} K_{i}=\frac{P_{i}^{-}}{P_{i}^{-}+R} \end{array}$$

(8) $$\begin{array}{c} {\hat{x}}_{i}={\hat{x}}_{i}^{-}+K_{i}*\left(x_{i}-{\hat{x}}_{i}^{-}\right) \end{array}$$

(9) $$\begin{array}{c} P_{i}=\left(1-K_{i}\right)*P_{i}^{-} \end{array}$$

Following this implementation, in order to obtain the filter’s result at any given time point *i*, and hence the prediction of the time series *x* at time *i*, one simply queries ${\hat{x}}_{i-1}$. Please note that the result of the filter is equivalent to consider that the time series under analysis is oscillating around a true value, which we can only observe in a noisy way. ${\hat{x}}_{i-1}$ thus represent such hidden average value, supposing the stationarity of the time series.

#### 2.2.5. Validation Models

If the previously described models are expected to yield good predictions of the future temperature, it is still necessary to validate them, i.e., demonstrating that the forecast is above random chances, and comparing the relative gain in information by them provided. To obtain a baseline, two validation models are here considered:

- Last temperature value: the last recorded value is used as the future prediction, i.e., the temperature is supposed to be constant. $T_{t}$ is thus assumed equal to $T_{t-1}$. Please note that this would be the default information used by a device without forecasting capabilities.
- Average daily temperature: the expected future temperature is assumed to be the average of the period to which it belongs to. In other words, $T_{t}=1/8\sum_{i=1}^{8}T_{t-i}$.

By comparing the model predictions with these ones, it is possible to assess the increment in the forecast precision, and compare this with the increase in the computational cost.

## 3. Results

Once the six methods for forecasting the ambient temperature in IoT devices have been defined (including the two validation models), this Section focuses on their validation by analysing the results they yield for the two data sets presented in Section 2.1.

To assess and compare the error of each method, we used the Mean Squared Error MSE metric, defined as:

(10) $$MSE=\frac{1}{N}\sum_{i=1}^{N}{\left(T_{i}-{\hat{T}}_{i}\right)}^{2},$$

*N* being the data set size, $T_{i}$ the observed (real) temperature at time *i*, and ${\hat{T}}_{i}$ the predicted value. MSE is, therefore, a risk function that allows to understand how accurate a predictor is. The higher the value of the MSE, the worst the predictor. Additionally, the expected average error of each model can be approximated as $\sqrt{MSE}$.

In what follows, results are organised in four parts. Firstly, we discuss the results obtained from the application of the models to the data that originated from the refrigerator (Section 3.1), for then presenting similar results for the temperatures in Lisbon (Section 3.2). Afterwards, both approaches are combined in Section 3.3, and their computational cost analysed in Section 3.4.

### 3.1. Results for the Indoor Temperature

**Figure 3:**
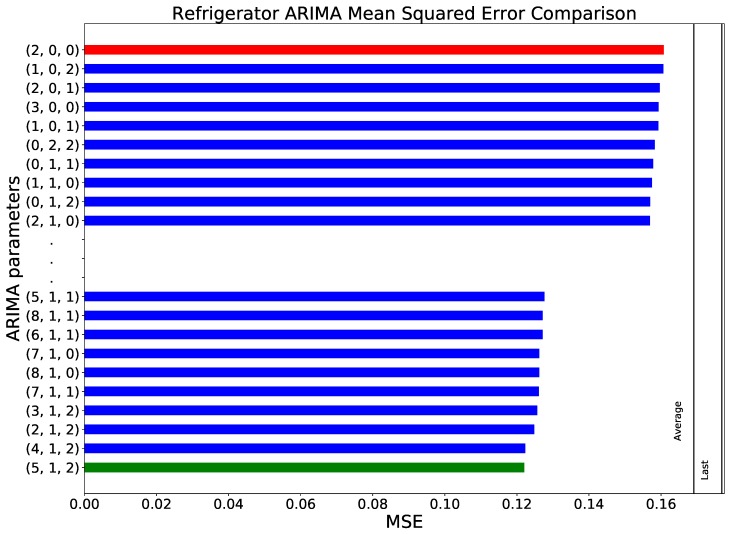
Comparison between different parametrisations of the ARIMA model, in terms of the MSE of the forecast, when applied to the refrigerator’s data set. The red and green bars respectively indicate the worst and best set of parameters. Additionally, the two vertical lines represent the values yielded by the two validation methods, i.e., the last and average temperature.

As a first step, it is necessary to optimise the parameters of the ARIMA model to minimise its error in the prediction. As discussed in Section 2.2.3, multiple ARIMA models were generated and applied to the data, in order to find the most suitable values for the parameters *p*, *d* and *q*. The graph shown in Figure 3 presents the results from this initial study, by depicting the MSE obtained for each set of (p,d,q) parameters. Please note that only those models that behave better than the validation models are shown, and the best and the worst of them are highlighted in green and red, respectively. Two conclusions can be drawn. Firstly, that the sensitivity of the model to these parameters is fairly low, as the best and worst parametrizations yield a MSE of respectively 0.122 and 0.161. Secondly, the best results are obtained for high values of the autoregressive order *p*, combined with an integration order *d* of one.

**Figure 4:**
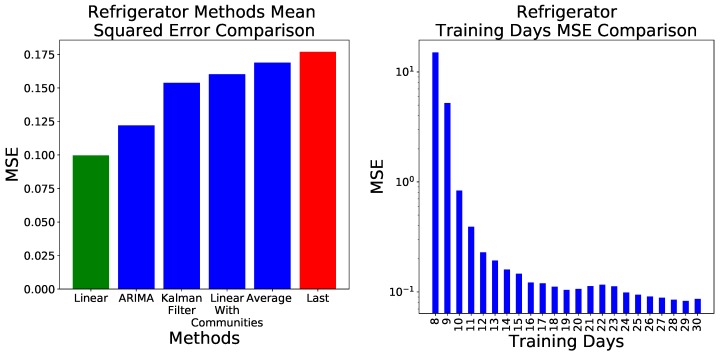
(**Left**) Error, measures as MSE, obtained by the six considered forecasting methods, when applied to the refrigerator’s temperature data set. The red and green bars respectively indicate the worst and best prediction models. (**Right**) Evolution of the MSE, as obtained by the linear model, as a function of the number of days used in the training.

Once the best ARIMA parametrization was established, specifically the (5,1,2) one, we compared the six forecasting methods previously described. Using the same approach of Figure 3, in Figure 4 Left we report the MSE obtained by each model. As opposed to what observed in Figure 3, the gap between the best and the worst model is larger than just a few decimals. Results span from 0.0996 of the basic linear model, to the 0.1602 of the linear model with the network-based feature selection. This compares to the 0.1689 and 0.1768 MSE obtained by the two validation models, i.e., the daily average and the last temperature value, respectively. Additionally, Figure 4 Right reports the evolution of the MSE as a function of the number of days used for the training of the linear model. It can be appreciated that 30 is a conservative value, and that similar performances can be obtained for as low as 19 days.

### 3.2. Results for the Outdoor Temperature

**Figure 5:**
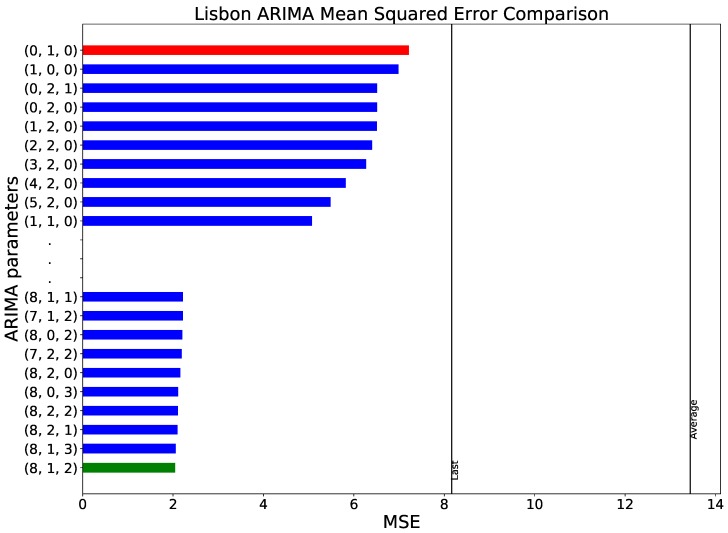
Comparison between different parametrisations of the ARIMA model, in terms of the MSE of the forecast, when applied to the Lisbon’s temperature data set. The red and green bars respectively indicate the worst and best set of parameters.

**Figure 6:**
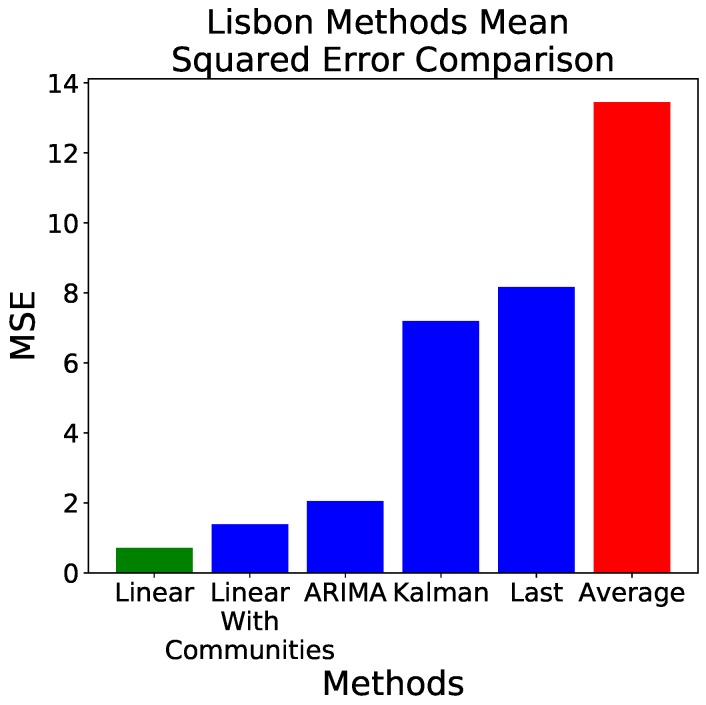
Error, measured as MSE, obtained by the six considered forecasting methods, when applied to the Lisbon’s temperature data set. The red and green bars respectively indicate the worst and best prediction models.

As previously introduced, all analyses shown for the evolution of the indoor temperature have been repeated for the time series corresponding to the city of Lisbon’s outside temperature. Consequently, Figure 5 and Figure 6 are constructed in the same way, and represent the same information as Figure 3 and Figure 4.

In the optimisation of the ARIMA parameters, depicted in Figure 5, one can note that similar results are obtained, with lowest prediction scores associated with high values of the autoregressive order *p*. The main difference resides in the scale of the error: while, in the case of the refrigerator temperature, all models scored below 0.16 MSE, when predicting Lisbon’s temperature the range increases to 2–8 MSE. This suggests that forecasting the ambient temperature is a more complex task than predicting the indoor one—a topic that will be discussed in Section 4.

When comparing all models, including the ARIMA with parameters (8,1,2), the ranking of models is similar to the one previously obtained—see Figure 6. It has nevertheless to be noted that, as opposed to the refrigerator’s example, when predicting Lisbon’s temperature the three models (ARIMA, and linear with/out communities) behave much better than the validation ones (being the exception the Kalman Filter, which scored similarly to one of the validation models), with a reduction of the MSE error of almost 14 orders of magnitude between the average and the linear model.

### 3.3. Sharing Information among Devices

As previously discussed, the cornerstone of the IoT paradigm is the possibility of sharing information among different devices, thus enabling the integration of different perceptions on the environment in which they are located. In the context here considered, one can envision a scenario in which the refrigerator is connected to internal and external devices and receives from them additional information. Such information could then be combined with the internal one, in order to construct a more complex forecasting model. In other words, thanks to the IoT paradigm, the refrigerator would be able to sense the world beyond its more immediate neighbourhood.

To simulate such scenario, we here consider the following extension of the linear model presented in Section 2.2.1:

(11) $$T_{t}=\beta_{0}+\beta_{1}T_{t-1}+\ldots +\beta_{9}T_{t-9}+\beta_{E}\left(E_{t-1}-E_{t-2}\right)+\beta_{r}R_{t}+\beta_{h}H_{t}+\epsilon ,$$

$E_{t}$ being the external temperature recorded at time *t*; $R_{t}$ a binomial variable whose value is one if it is raining at time *t* (and zero otherwise); and $H_{t}$ a second variable whose value is one if *t* is bank holiday. Please note that the first new term represents the evolution (mathematically, the discrete derivative) of the external temperature; the second additional information about the external environment, also obtained through www.wunderground.com; and the last one information about the usage of the building. The model is then able to include information about the external dynamics, and adapt its forecast in cases of strong external perturbations, e.g., rapid temperature drops, reduced activity in the building, and so forth.

When the model of Equation (11) is trained and compared with the original linear model, the effect is a modest reduction in the forecast error, from 0.0996 to 0.0865. While in this case the improvement is of only a 1.15%, it is enough to shed light on the potential usefulness of an ecosystem of interconnected devices.

### 3.4. Computational Cost

**Figure 7:**
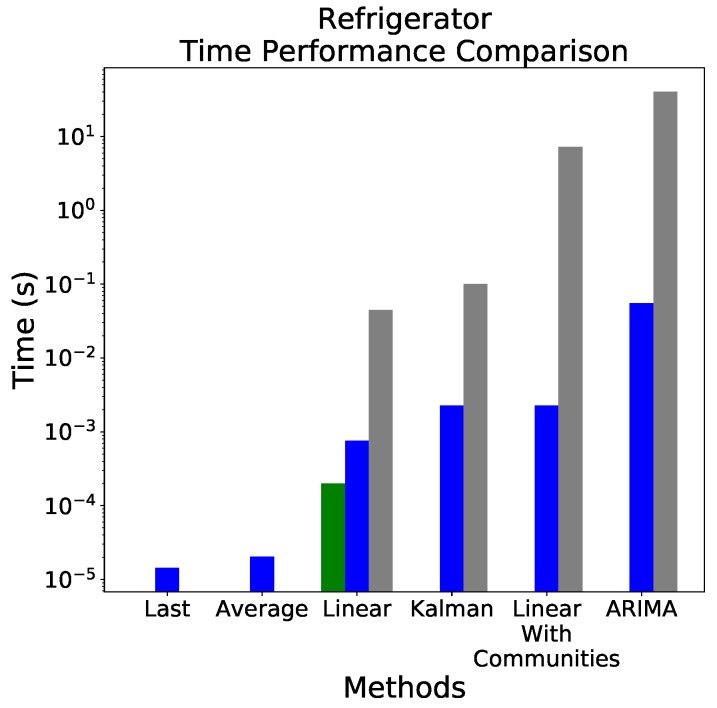
Time, in seconds, each method took to be trained (grey bars) and forecasting (blue bars) for the refrigerator data set. The (only) green bar refers to the Arduino Uno forecasting tests performance. The values refer to a total of 100 consecutive executions of the same operation.

As a last issue, the previous results must be complemented with an analysis of the computational cost of each forecasting model, as, due to the limited power available in most IoT devices, a compromise between precision and requirements may be needed. Consequently, Figure 7 reports the time required both for training (grey bars) and applying (blue bars) each algorithm, as calculated in a 5th generation Intel^®^ Core^™^ i7-5500U CPU and implemented in Python 3.6. Please note that here we are only interested in the relative magnitude of the computational cost, and not in the exact value—which would strongly depend on the on-board hardware and software. Also note that, in the case of the ARIMA model, the cost of optimising the model’s parameters has not been considered—we thus suppose that an optimal model has already been chosen, and that it does not change with time.

It can be appreciated that results are very heterogeneous, with the ARIMA model’s cost being one and three orders of magnitude higher than that of the linear (respectively, with and without communities) models. When considering that the simplest linear model was also the most precise (Figure 4 and Figure 6), it is easy to conclude that it should be the method of choice for the described scenario. If storing information about the last 30 days may suppose a memory problem, Figure 4 Right suggests that this can be mitigated by reducing the size of the training data set, and still get acceptable prediction errors. It is finally worth noting that the training cost can be avoided by supposing that the forecast model is constant - a condition which holds, provided the indoor heating/cooling logic is not changed. The parameters of the model can then be passed to the refrigerator by an external system; or alternatively, be calculated by the refrigerator itself with a low frequency, e.g., once per week or per month.

As an IoT application will seldom be constructed upon a laptop hardware, one last question is related to how the figures presented in Figure 7 translate to a real application. To compare, a first-generation Raspberry Pi hardware, one of the most commonly used platforms for testing IoT concepts, yields a computational power of 0.065 GFLOPS, roughly 2% of that of an i7-5500U CPU (3.61 per core). Supposing a conservative estimation of a ×100 slow-down, this still implies that a prediction with the linear model can be executed in less than 0.1 s in a Raspberry Pi, still enough to support any application. On the other hand, considering many of present-day IoT devices are embedded with 8-bit devices, as it is the case of the Arduino, the algorithm that presented best results (Linear) was also uploaded to an Arduino Uno to understand how it behaved, computationally speaking. This cannot be referenced without noting that, in such devices, the algorithm should not be trained in-loco, but, rather, trained on a different machine and then uploaded to the device where it will perform. In fact, it was impossible, with the limited memory of the specific Arduino model that was used, to train the model.

That being said, the Linear algorithm coefficients were calculated and the model built upon those in the Arduino Uno. Then, the same method for calculating the forecasting performance (running 100 consecutive executions) was executed on the device and the elapsed time calculated (this result can be seen in Figure 7—the green bar on the Linear index). As it can be observed in the aforementioned figure, the Arduino Uno performs quite well on forecasting values using the provided model. In fact it is even faster than the CPU used for this work. This is influenced, of course, by the fact that, in the Arduino, the model is nothing more than simple Algebra, calculated directly by the processor of the device whereas, in the CPU, a whole software stack must be considered, all the way from the programming language to the Operating System. Yet other metrics worth noting are the fact that the simple Arduino program occupied no more than 190 bytes (9%) of dynamic memory of a total of 2048 bytes available. Regarding program storage space, it occupied 1690 bytes (5%) of a maximum of 32,256 bytes available, as provided by the Arduino IDE.

Additionally, IoT hardware has seen an important improvement in the last few years, being easily possible to reduce this time by one order of magnitude [33]. We can therefore conclude that the computational cost will not be a major issue in the application here presented.

## 4. Discussion and Conclusions

In this work we have discussed the feasibility of an IoT scenario composed of an home appliance (specifically, a refrigerator) integrating information from own and external sensors, and performing a forecast on the ambient temperature. Several forecast models have been compared, both in terms of their precision and computational cost. From a global point of view, results indicate that such scenario is technically feasible, and that the refrigerator could use models to integrate information and generate short-term temperature forecasts without the need for specific hardware. While the computational cost has been calculated in a standard computer, it is nevertheless small enough to not pose a problem—even supposing a reduction in the computational speed of three order the magnitudes, the time required for a prediction is still of the order to a second. Also, it is worth noting that the performance of the best algorithm is remarkable, being able to predict the temperature within a temporal horizon of 2 and half hours with an error of ≈0.09 °C. From a more specific perspective, several conclusions can be drawn, which we discuss below.

First of all, the scenario here described should be understood beyond the specific problem of forecasting the temperature inside a building. It is instead representative of the general problem of integrating and processing information in an IoT environment, with the limitations (e.g., computational power, memory, or bandwidth) typical of IoT devices. The specific data mining task, i.e., forecasting the temperature, is also archetypical of an IoT application: a *prima facie* simple problem, whose solution can open novel applications, including, in this case, the participation in energy markets and arbitrage.

Secondly, the problem of the short-term temperature forecasting is an interesting one. The most simple model, just including a weighted linear combination of past data, is able to outperform more complex approaches, including feature selection strategies or ARIMA models. When compared with the average daily temperature, as would be calculated by a non-intelligent device, this model is able to reduce the error by two third. Additionally, the MSE when forecasting the indoor temperature resulted substantially lower than the one for the outdoor temperature. This seems to suggest that the dynamics of the cooling/heating system of a building is more simple, or at least more predictable, than that of Nature. When these results are combined, one can conclude that this specific data mining task is especially suitable to be prototyped in an IoT environment.

Digging deeper in the IoT paradigm, we have studied the possibility of merging information from several sources; in this case, we supposed the refrigerator was also getting information about the external weather and the building usage from external devices. Due to the way the temperature is recorded and processed, i.e., just nine values per day, the improvement associated with the use of the external temperature has been marginal—the time between subsequent measurements is probably too large to describe sudden changes in the external environment. Also, the discrimination between working days and holidays yielded minimal improvements—as the heating/cooling of the building is kept constant throughout the year. Despite this, this study reveals that several improvements can be foreseen. First of all, one can consider increasing the temporal resolution of both external and internal temperature measurements, which may possibly increase the forecasting capabilities of the system. Please note that such resolution is not arbitrary, but instead a constrain of the specific hardware considered, and cannot at present be changed by the user. Secondly, it is possible to foresee a scenario in which the external device forecasts the future external temperature, using one of the tailored available algorithms [21,22,23,24,25], for then transmitting this information to the refrigerator. In principle, this would allow to obtain a more precise indoor forecast, as it would include the expected future (as opposed to past) variation in the external temperature.
